# Hard clam walking: Active horizontal locomotion of adult *Mercenaria mercenaria* at the sediment surface and behavioral suppression after extensive sampling

**DOI:** 10.1371/journal.pone.0173626

**Published:** 2017-03-09

**Authors:** Stephen T. Tettelbach, James R. Europe, Christian R. H. Tettelbach, Jason Havelin, Brooke S. Rodgers, Bradley T. Furman, Marissa Velasquez

**Affiliations:** 1 Department of Biology, LIU-Post, Brookville, New York, United States of America; 2 Southeast Fisheries Science Center, NOAA, Miami, Florida, United States of America; 3 College of Natural Resources, University of California Berkeley, Berkeley, California, United States of America; 4 Marine Program, Cornell Cooperative Extension of Suffolk County, Southold, New York, United States of America; 5 School of Marine & Atmospheric Sciences, Stony Brook University, Southampton, New York, United States of America; 6 Department of Environmental Sciences, University of Virginia, Charlottesville, Virginia, United States of America; 7 Department of Biology, Cornell University, Ithaca, New York, United States of America; University of California, UNITED STATES

## Abstract

Locomotion of infaunal bivalve mollusks primarily consists of vertical movements related to burrowing; horizontal movements have only been reported for a few species. Here, we characterize hard clam walking: active horizontal locomotion of adults (up to 118 mm shell length, SL) of the commercially important species, *Mercenaria mercenaria*, at the sediment surface—a behavior only briefly noted in the literature. We opportunistically observed walking over a 10-yr period, at 9 different sites in the Peconic Bays, New York, USA, and tested several hypotheses for the underlying cause of this behavior through quantitative field sampling and reproductive analyses. Hard clam walking was exhibited by males and females at equal frequency, predominantly during June/July and October, when clams were in peak spawning condition. Extensive walking behavior appears to be cued by a minimum population density; we suggest it may be mediated by unidentified pheromone(s), infaunal pressure waves and/or other unidentified factors. There was no directionality exhibited by walking clams, but individuals in an area of extensive walking were highly aggregated and walking clams were significantly more likely to move toward a member of the opposite sex. Thus, we conclude that hard clam walking serves to aggregate mature individuals prior to spawning, thereby facilitating greater fertilization success. In the process of investigating this behavior, however, we apparently oversampled one population and reduced clam densities below the estimated minimum threshold density and, in so doing, suppressed extensive walking for a period of >3 years running. This not only reinforces the importance of detailed field investigations of species biology and ecology, even for those that are considered to be well studied, but also highlights the need for greater awareness of the potential for research activities to affect focal species behavior.

## Introduction

Locomotion and/or seasonal migration are integral to the survival of individual animals, as well as populations. These processes have been extensively studied in terrestrial fauna [1] and aquatic vertebrates [2] in the contexts of feeding, reproduction and avoidance of predators, competitors or suboptimal abiotic conditions. In general, less is known about locomotory behavior of marine invertebrates.

In the Phylum Mollusca, the Cephalopoda (including squids and octopuses) are renowned for their locomotory abilities [3]; in other molluscan classes, locomotion usually occurs at a snail’s pace or, in the case of most Bivalvia, even more slowly [4]. Active movement of infaunal bivalves is primarily vertical and is related to burrowing in the sediment [5]–often in response to tidal or seasonal cues [6]. Several exceptions have been described: the epibenthic Pectinidae (scallops) and Limidae (file shells) are capable of short bursts of swimming [7], while leaping is prominent in semi-infaunal Cardiidae (cockles) and Asaphidae as well as infaunal razor clams (Solenidae) and Mactridae [8–11]. These forms of locomotion are thought to be used primarily to escape would-be predators [12].

Crawling is exhibited by other bivalve taxa, particularly among epibenthic marine mussels, Mytilidae [13] and infaunal pearly freshwater mussels, Unionida [14–17]. In mytilids, vertical and horizontal crawling enables mussels to avoid smothering by conspecifics or surficial sediments [18]. Crawling may also serve to aggregate mytilids, which in turn may increase rates of survival by reducing dislodgement [18,19] or desiccation [20]. Horizontal movement at the sediment surface by Unionids, however, is believed to serve the purpose of aggregating reproductively mature individuals in preparation for spawning [15,17]. The process of aggregation has been shown to increase the probability of successful fertilization in free-spawning invertebrates, including *Mercenaria mercenaria* [21–25]. In *Limecola* (*Macoma*) *balthica*, crawling at the sediment surface has been attributed to the need to optimize deposit-feeding by stunted individuals [26] or to a heavy load of parasitic trematodes [27]; in the latter case, crawling did not appear to provide any particular benefit to the clams themselves.

The hard clam or quahog, *Mercenaria mercenaria*, is a commercially important and extensively studied member of the Veneridae, a family in which crawling at the sediment surface is well known in recently settled plantigrades (< 1 mm) [28–30]. Active horizontal crawling of 2–3 mm *Mercenaria* has also been observed, with clams moving as rapidly as 32 mm in 15 min, partly while buried and partly while at the sediment surface [28]. However, in each of two larger size classes (28 and 41 mm) of hard clams, only ~17% moved during a 38 d experiment; larger clams moved laterally an average distance of ~55 mm, twice as far as smaller clams [28]. Horizontal crawling by larger quahogs was considered to be unimportant and it was concluded that these clams essentially lead a sedentary life [28]. While substantial passive, lateral displacement of large juvenile/small adult *Mercenaria* by storms [31] and tidal erosion [32] have been documented, small scale lateral movements by large hard clams have rarely been reported. *Mercenaria* planted in enclosures were noted to move laterally [33,34] while this phenomenon was also observed, infrequently, during studies of vertical positioning [6]. None of the above authors reported if lateral movements of large *Mercenaria* were active, nor was any purpose suggested for this type of locomotion.

Here, we provide detailed evidence (including video) of active horizontal locomotion of adult (up to 118 mm shell length, SL) *Mercenaria mercenaria* (hereafter ‘hard clam walking’), whereby epibenthic clams (with the shell protruding visibly from the sediment surface: [15]) were directly observed to move laterally at the sediment surface and/or were seen at the end of freshly excavated trails. We characterized walking behavior and the sites where we have documented it over a 10 yr period and tested several hypotheses for the underlying causes of this type of locomotion. As hard clam walking may represent either a stress response or an adaptive behavior to increase the probability of survival, growth or reproduction, we examined two sets of questions: (1) what factors might affect specific metrics of hard clam walking behavior: e.g., effects of environment (season, directionality of tidal flow) and clam condition (size, gender) on the occurrence and nature of observed trails and (2) what factors might differ between areas where extensive hard clam walking was and was not observed: e.g. environmental (sediment type, food levels), clam condition (size, gender, reproductive state, incidence of parasitism) and population parameters (density, nearest neighbor (NN) distance, degree of aggregation). Further, we discuss how, and why, walking behavior has apparently been suppressed at one site by our extensive scientific sampling.

## Materials and methods

### Ethics statement

As both a bivalve mollusk and a commercially and recreationally harvested species, *Mercenaria mercenaria* is not subject to any regulations with regard to experimentation or care/welfare. In public New York state waters (which includes Orient Harbor and other areas where field work was conducted) harvest of 100 clams per day is allowed without a license [35]; we also hold a License to Collect or Possess: Scientific (#1661) from the New York State Department of Environmental Conservation (issued to Stephen Tettelbach). No other species (protected or otherwise), besides *Mercenaria*, were sampled during this study.

### Opportunistic field observations

Initial observations of *Mercenaria mercenaria* locomotion were made serendipitously on 1 June 2007 while diving to examine whelk populations at a site in Gardiners Bay, New York (N 41° 06.36’ W 72° 16.32’: Table 1). Here, more than two dozen epibenthic clams (~40–80 mm in shell length (SL)) were seen at the end of 30–60 cm trails. On 8 June 2010 (while diving to survey bay scallop populations: [36]) at a site ~100–200 m off the shoreline of East Marion, New York (center = N 41° 07.694’ W 72° 19.624’); we again saw numerous clams at the end of trails and directly observed clams walking. We began to more fully document the phenomenon on this date and on subsequent dives at this ‘walking area’ on 9 and 11 June, and 1 and 19 July 2010, and periodically each year until 2016. We also surveyed another area (center = N 41° 07.729’ W 72° 19.610’), ~50–60 m to the NNE of the aforementioned area, which was designated as ‘non-walking’, as repeated sampling here over 6 yr only revealed active clam walking (n ~5 total) on only two dates.

**Table 1:** Opportunistic observations of actively walking and non-walking epibenthic hard clams, *Mercenaria mercenaria*, made while diving in the Peconic Bays, New York, USA, June 2007 –June 2016.

	Water Temp		Depth (m)		
Date	Sur (Bot) (°C)	Site (Area)/GPS Center	at MLW	Sediment	# clams
1 Jun 2007	18.3	Gardiners Bay (a): 41° 06.36’ N 72° 16.32’ W	6.5	MS/Sh	>24
24 Jul 2007	~22	Gardiners Bay (b): 41° 02.448’ N 72° 16.701’ W	2–3	S/G/C/B	>6
23 Apr 2008	~13.3	Orient Harbor (PN-1): 41° 07.700’ N 72° 17.513’ W	1.3–1.5	S/Mac	1
16 Aug 2008	25.6	Great Peconic Bay: ~40° 59.412’ N 72° 29.602’ W	1–2	S/Mac	~2–3
4 May 2009	~15.8	Hallock Bay (a): ~41° 07.876’ N 72° 16.066’ W	3–4	M/Sh/Cf/Mac	>10
18 May 2009	~13.6	Orient Harbor (EM-w): 41° 07.725’ N 72° 19.641’ W	1.3–1.7	S/Mac	>6
8,9,11 Jun 2010	19.4–20.6	Orient Harbor (EM-w): 41° 07.711’ N 72° 19.623’ W	1.3–1.7	S/Sh/G/Mac	79
	(19.3–19.9)				
1 Jul 2010	~23.0	Orient Harbor (EM-w): 41° 07.658’ N 72° 19.641’ W	1.3–1.7	S/Sh/G/Mac	5–6
19 Jul 2010		Orient Harbor (EM-w): 41° 07.666’ N 72° 19.646’ W	1.3–1.7	S/Sh/G/Mac	23
11 Oct 2010	17.1	Orient Harbor (EM-w): 41° 07.724’ N 72° 19.632’ W	1.3–1.5	S/Sh/G/Mac	3
22 Oct 2010	13.6	Southold Bay: 41° 03.964’ N 72° 24.201’ W	1.5–2.5	MS/Sh/Cf	1
2 May 2011	14.8	Orient Harbor (EM-w): 41° 07.713’ N 72° 19.645’ W	1.3–1.7	S/Sh/G/Mac	4
21 Jun 2011		Orient Harbor (EM-w): 41° 07.714’ N 72° 19.596’ W	1.3–1.7	S/Sh/G/Mac	≥6†
12 Oct 2011	18.2 (18.3)	Orient Harbor (EM-w): 41° 07.712’ N 72° 19.637’ W	1.3–1.7	S/Sh/G/Mac	≥8
15 May 2012	16.0 (15.8)	Orient Harbor (EM-1): 41° 07.613’ N 72° 19.597’ W	1.5–2	S/Sh/G/Mac	6
7,11,12 Jun 2012	19.4–21.2	Orient Harbor (EM-w): 41° 07.701’ N 72° 19.631’ W	1.3–1.7	S/Sh/G/Mac	>16
	(20.1–21.2)				
20 Jun 2012	21.7	Orient Harbor (EM-w): 41° 07.701’ N 72° 19.631’ W	1.3–1.7	S/Sh/G/Mac	>6
20 Jun 2012	21.7	Orient Harbor (EM-nw): 41° 07.729’ N 72° 19.610 W	1.3–1.7	S/Sh/G/Mac	1
3 Aug 2012	25.1 (24.5)	Orient Harbor (EM-w): 41° 07.701’ N 72° 19.631’ W	1.3–1.7	S/Sh/G/Mac	3–4
3 Aug 2012	25.1 (24.5)	Orient Harbor (EM-nw): 41° 07.729’ N 72° 19.610 W	1.3–1.7	S/Sh/G/Mac	2
28 Aug 2012	25.6	Orient Harbor (EM-w): 41° 07.701’ N 72° 19.631’ W	1.3–1.7	S/Sh/G/Mac	3–4
28 Aug 2012	25.6	Orient Harbor (EM-nw): 41° 07.729’ N 72° 19.610 W	1.3–1.7	S/Sh/G/Mac	~3
20 Sep 2012	21.1 (21.1)	Orient Harbor (EM-w): 41° 07.696’ N 72° 19.630’ W	1.3–1.7	S/Sh/G/Mac	10
13 May 2013	16.7	Greenport Harbor (a): ~41° 05.692’ N 72° 20.885’ W	3	M-MS/Cf/Sh	1*
13 May 2013	15.1	Orient Harbor (EM-w): 41° 07.701’ N 72° 19.631’ W	1.3–1.7	S/Sh/G/Mac	1*
5 Jun 2013	19.2	Orient Harbor (EM-w): 41° 07.701’ N 72° 19.631’ W	1.3–1.7	S/Sh/G/Mac	~6
11 Jun 2013	20.0	Northwest Harbor: 41° 00.873’ N 72° 15.389’ W	3	S/Mac	1
15 Jul 2013	25.7	Orient Harbor (EM-w): 41° 07.701’ N 72° 19.631’ W	1.3–1.7	S/Sh/G/Mac	4
15 Jul 2013	25.7	Orient Harbor (EM-nw): 41° 07.729’ N 72° 19.610 W	1.3–1.7	S/Sh/G/Mac	5
18 Jul 2013	21.7	Gardiners Bay (c): 41° 04.032’ N 72° 04.807 W	3	S/Sh/B/Mac/E	1
22 Jul 2013		Orient Harbor (EM-w): 41° 07.701’ N 72° 19.631’ W	1.3–1.7	S/Sh/G/Mac	4
22 Jul 2013		Orient Harbor (EM-nw): 41° 07.729’ N 72° 19.610 W	1.3–1.7	S/Sh/G/Mac	~8
7 Aug 2013	24.3	Orient Harbor (EM-w): 41° 07.701’ N 72° 19.631’ W	1.3–1.7	S/Sh/G/Mac	10
7 Aug 2013	24.3	Orient Harbor (EM-nw): 41° 07.729’ N 72° 19.610 W	1.3–1.7	S/Sh/G/Mac	2
7 Aug 2013	23.9	Greenport Harbor (b): 41° 05.823’ N 72° 20.819’ W	~2.5–6	MS/Cf/Sh/Mac	1
27 Aug 2013	23.9	Greenport Harbor (c): 41° 05.848’ N 72° 20.769’ W	~3–8	MS/Cf/Sh/Mac	3
20 May 2014	18.9	Hallock Bay (b): 41° 08.251’ N 72° 16.396’ W	1.7	MS/Mac	1
29 May 2014	16.7	Orient Harbor (EM): 41° 07.714’ N 72° 19.641’ W	1.3–1.7	S/Sh/G/Mac	1
22 May 2015	15.6	Orient Harbor (EM-nw): 41° 07.729’ N 72° 19.610 W	1.3–1.7	S/Sh/G/Mac	2
10 Jun 2015	18.6	Orient Harbor (EM-w): 41° 07.708’ N 72° 19.637’ W	1.3–1.7	S/Sh/G/Mac	3
29 Jul 2015	26.7	Orient Harbor (G): 41° 06.347’ N 72° 20.892’ W	3.5	MS/Sh/Cf	1
12 Aug 2015	25.6	West Neck Creek: 41° 03.856’ N 72° 21.291’ W	1.3–1.7	P/S	1
15 Oct 2015	17.3 (17.3)	Orient Harbor (EM-w): 41° 07.717’ N 72° 19.619’ W	1.3–1.7	S/Sh/G/Mac	~3
15 Oct 2015	17.3 (17.3)	Orient Harbor (EM-nw): 41° 07.728’ N 72° 19.613’ W	1.3–1.7	S/Sh/G/Mac	~3
27 May 2016	15.8	Orient Harbor (PN-2): 41° 07.701’ N 72° 17.465’ W	1.3	S-MS/Sh/G/Mac	7
3 Jun 2016	20.6	Orient Harbor (PN-3): 41° 07.697’ N 72° 17.485’ W	1.3	S-MS/Sh/G/Mac	2
10 Jun 2016	18.4	Orient Harbor (PN): 41° 07.663’ N 72° 17.416’ W	1.3	S-MS/Sh/G/Mac	1
10 Jun 2016	18.0	Orient Harbor (EM-w): 41° 07.701’ N 72° 19.631’ W	1.3–1.7	S/Sh/G/Mac	1

Turquoise shaded text = confirmed active walking: via direct observation and/or with recent trail; grey shaded text = non-walking (no trail), but in walking position (siphons parallel to sediment surface); not highlighted = only non-walking clams: completely out of sediment, laying on side—or—shell visible above sediment surface, with siphons facing upward, perpendicular to sediment surface. Only the most active form of behavior is denoted for a given date. Water temperature (sur = surface; bot = bottom), Site (Area)/GPS: ~: estimated from nearby site, or Google Earth, respectively. Sites are locations separated by 100’s– 1000’s of m; a, b, c = different sites within 1 embayment. Areas within sites (-*x*) are separated by 10’s of m. Orient Harbor sites: PN = off Peter’s Neck; EM = off East Marion (EM-w and EM-nw = ‘walking’ and ‘non-walking’ areas); G = Greenport. Depth: MLW = mean low water. Qualitative sediment type: S = Sand, M = Mud, MS = muddy sand, Sh = shell, G = gravel, C = cobble, B = boulders, P = peat, Mac = >1% cover of macroalgae, E = eelgrass, Cf = live *Crepidula fornicata*. # clams = # of *Mercenaria* exhibiting the behavior denoted:

^†^ = 1 individual appeared to release gametes;

* = deep walker (shell not visible at sediment surface).

Additional opportunistic observations of clam walking behavior were made by the authors between 1 June 2007 and 10 June 2016, during more than 700 dives (most to monitor bay scallop populations). Walking was observed at 9 of 15 different sites where we noted epibenthic hard clams in the Peconic Bays; site characteristics, including depth, qualitative sediment type and water temperature were also noted (Table 1).

### Formulation of hypotheses

Immediately after we observed hard clam walking, we began to formulate hypotheses about what factors might be driving this behavior. Initial ideas were refined after reviewing pertinent literature and we formalized data collection/analyses to answer the two sets of questions noted above and detailed in Tables 2 and 3. These activities took place at various times during the study period.

**Table 2:** Summary of factors hypothesized to affect specific metrics of observed hard clam walking behavior in the Peconic Bays, New York, USA.

Factor	Walking Metric	Refs	Statistical Test(s)	Outcome
shell length, gender	shell ht above sediment	[52]	1-way Ancova,	NS
gender			1-way Anova	NS
shell length, gender	furrow depth		1-way Ancova,	NS
gender			1-way Anova	NS
season	trail length	[14,15]	t-test,	NS
shell length			1-way Ancova,	NS
gender			1 way Anova	NS
tidal flow at site	trail heading	[28,32]	χ^2^	NS
nearest epibenthic	gender pairs:	[72]	χ^2^	NS
neighbor in any direction	1 or both			
from actively walking	active walkers			
clam				
nearest epibenthic	gender pairs:	[72]	χ^2^	*
neighbor in 120° forward	1 or both			
direction from actively	active walkers			
walking clam				

Statistical test outcomes: NS = not significant (p > 0.05);

* = p < 0.05,

** = p < 0.01,

*** = p < 0.001.

**Table 3:** Summary of factors hypothesized to differ between adjacent (~50–60 m apart) areas at the East Marion site where hard clam walking was frequently observed (‘walking area’) versus rarely observed (‘non-walking’ area).

Factor	References	Statistical Test	Outcome
food level: total chl a	[26]	t-test	NS
food level: chl a (>5 μm)	[26, 44]	t-test	NS
sediment grain size	[40,41,42,51,52]	t-test	NS
gender	[15,23]	χ^2^	NS
shell length	[15,16,23,54]	2-way Anova	NS
reproductive condition	[15,44,45]	Fisher Exact test	NS
parasite load	[27]	Fisher Exact test	NS
density: total		1-way Anova	***
density: epibenthic only	[23]	1-way Anova	***
degree of aggregation	[15,17,21–24,49,54]	χ^2^	***
NN: epi- or endobenthic	[47,49,50]	1-way Anova	***
NN: epibenthic only	[23,47,49,50,72]	1-way Anova	***

NN = nearest neighbor distance. Statistical test outcomes: NS = not significant (p > 0.05);

* = p < 0.05,

** = p < 0.01,

*** = p < 0.001.

### Characterization of hard clam walking behavior

Walking behavior was characterized through *in situ* observations, photographs and videos taken primarily at the East Marion site. Still photographs (5 megapixel resolution) were taken of clams with recent (i.e. ‘clean’) trails behind their shells to characterize their position/orientation, while video footage (in June of 2010–2012) was taken of clams which were directly observed to walk. Video footage of 9 different clams (10 total walks) was subsequently analyzed to characterize mechanics of locomotory behavior and to quantify the duration, distance and time interval between individual walks.

*In situ* measurements were also made of: clam shell height above the sediment (n = 59), depth of the trail (furrow) made by walking clams (n = 50), trail length, trajectory and direction of travel, via a wrist-mounted dive compass (n = 102, 82, and 79, respectively). A t-test was done to compare length of trails in June/July versus October. This was nonsignificant and so pooled data were used in an Ancova to examine whether trail length varied with clam gender, with shell length (SL) as a covariate. The effects of clam gender on shell height above the sediment and furrow depth were also investigated in this way. In cases where SL was a nonsignificant covariate, respective 1-way Anova’s were run to examine the effect of gender on the response variable. Directionality of travel was examined via a χ^2^ goodness of fit test for circular data [37], with heading direction categorized into 45° intervals (0–44°, 45–89°, etc.).

### Maximum duration of epibenthic habitation

Shell lengths (SL) of the largest epizoic *Crepidula fornicata* found on epibenthic clams were measured, and calculated *C*. *fornicata* growth rates [38] were used to estimate the maximum length of time that clams may have remained on the sediment surface; these estimates were compared to our observations of *C*. *fornicata* growth on shells of live bay scallops, *Argopecten irradians irradians*.

### Comparison of adjacent ‘walking’ and ‘non-walking’ areas

#### Relative areal sizes

At the East Marion site, hard clam walking was only observed within a limited area; dimensions of a portion of this ‘walking’ area were estimated on 20 September 2012 from positions of actively walking clams (n = 10) marked by divers with rebar stakes. GPS coordinates were then taken from surface floats tied to these stakes. Distances between perimeter points of the walking clam polygon were computed using the Haversine formula [39]; the area was calculated to be ~1230 m^2^. Based on the approximate locations where other walking clams were seen in this vicinity from 2010–2015, the overall ‘walking’ area was estimated to be ~3000 m^2^. The surveyed ‘non-walking’ area was ~1000–1500 m^2^.

#### Measurement of environmental factors

Surficial sediments in both of these areas were comprised of sand and sparse shell hash with ≤10% cover of live *Crepidula fornicata* and macroalgae; depth at mean low water (MLW) was 1.3–1.7 m. Grain size fractions of surface sediments were further characterized as this is known to affect burrowing rates of infaunal species [40] and the density of *Mercenaria* [41]. On 12 June 2012, replicate samples (n = 3 per area) of the top (1 cm) sediment layer were taken by divers and frozen upon return to the laboratory. Once thawed, course fractions (gravel and sand) were wet sieved and fine fractions (silt + clay) were determined using the pipette method [42]. Means of the proportion of sand in the ‘walking’ and ‘non-walking’ areas (n = 9 subsamples per area) were compared via t-test. Surface and bottom water temperature (see Results) and salinity (which ranged from 26.9–29.7 and 27.0–29.7 for surface and bottom water, respectively; not differentiated by area) were recorded on dates of field observations with a YSI Model 85 meter. Relative current flow [43] for the East Marion and Peter’s Neck sites was determined to be ‘low’ on 29–31 July 2013 [36].

Additional sampling in the ‘walking’ and ‘non-walking’ areas was done to measure total chlorophyll a (chl a) and the chl a fraction represented by phytoplankton cells ≥5 μm; the latter fraction represents suitable food for adult hard clams [44]. Water samples were taken ~7–15 cm above the sediment surface on 12 June 2012, on ebb and flood tides (3 hr before and 2.2 hr after slack low tide, respectively). Median total chl a and mean levels of the ≥5 μm fraction (n = 6 subsamples for each area) were compared via t-test.

#### Clam gender and condition

Clams were permanently removed from ‘walking’ and ‘non-walking’ areas at East Marion to examine gender, reproductive condition and parasitic load. Gender of walking and non-walking clams was determined on multiple dates via microscopic (40 to 100×) examination of gonadal tissue for the presence of eggs or sperm [44], while reproductive condition (stages D1-D2, S1-S4) of walking clams sampled on 19 July 2010 and 12 June 2012 was assessed via histology [45]. Frequencies of walking clams in spawning condition (stages S1-S4) on the latter date were compared for the ‘walking’ and ‘non-walking’ areas at East Marion via Fisher Exact Test. Parasite loads in visceral mass (including gonadal) tissues were assessed in the same histological slides; frequencies of individuals with parasites in the ‘walking’ and ‘non-walking’ areas, for both dates, were compared via Fisher Exact Test. A 2-way ANOVA was run to test whether ln clam shell length differed with respect to gender or location (‘walking’, ‘non-walking’ areas). χ^2^ tests, with Yates correction for continuity [37], were used to test whether gender frequency differed amongst walking and non-walking clams and whether the nearest epibenthic neighbors of actively walking clams were of the opposite gender in: (a) any direction, or (b) only in the forward direction (120°) of walking.

#### Population parameters

Total (epibenthic + endobenthic) and surface (epibenthic) clam densities were quantified between 2010–2016 in 1 m^2^ quadrats: (a) centered on actively walking epibenthic clams (n = 26 for total density, n = 32 for epibenthic density) at East Marion and Peter’s Neck, or placed haphazardly, after swimming for several m with eyes closed, in locations without actively walking clams in (b) the ‘walking’ area (n = 29 for each) and (c) ‘non-walking’ area (n = 26 for each) at East Marion. Quadrats were ≥ 2 m apart. Counts of epibenthic and endobenthic clams within each quadrat were made after surficial sediments were removed by fanning and/or probed by hand to a depth of ~15 cm. Total and surface clam densities (ln x+1) in the two areas were compared via respective 1-way ANOVA’s, followed by multiple comparisons tests.

Total (epi- + endobenthic) clam densities were also used to examine the nature of aggregation resulting from the walking behavior using Morisita’s Index (I_mor_: [46]). Distributions were classified as overdispersed (I_mor_ = 0), random (I_mor_ = 1) or aggregated (= contagious: I_mor_ > 1); significance was tested via χ^2^. I_mor_ is equivalent in most cases to ‘mean crowding’ [47] and was considered robust to the effects of sample size [48].

Nearest neighbor (NN) distances [49] were measured to the nearest cm from actively walking clams to other clams (epibenthic or endobenthic) within the quadrat (or outside, as necessary) or, in quadrats without walking clams, between endobenthic or non-walking epibenthic individuals. In cases where NN distances were not measured (n = 4, 18 for epi- + endobenthic, and epibenthic clams, respectively), they were estimated on the basis of clam densities quantified for respective dates/sites [50]. The use of 1 m^2^ quadrats was appropriate [47] based on the observed distances traveled by walking clams. Nearest neighbor distances (square root transformed) were compared via simple Type III ANOVA, with data categorized by sampling area designation (W: ‘walking’ area, WNW: ‘walking’ area with no walking clams observed, NW: ‘non-walking’ area).

## Results

### Opportunistic field observations

Active hard clam walking was observed at 9 different sites in the Peconic Bays, New York, at depths ranging from ~1.3–6.5 m below mean low water (MLW) and in a range of surficial sediment types (Table 1). At most of these sites, walking behavior was only exhibited by 1–2 individuals; however, many (5 to 79) clams were actively walking on specific dates at East Marion, Peter’s Neck and Gardiners Bay (Table 1). Other than single individuals observed on 23 April 2008 (13.3°C) and 22 October 2010 (13.6°C), active *Mercenaria* walking at the sediment surface was only observed between late May—October, at water temperatures from 15.8 to 26.7°C. Walking activity was most pronounced at East Marion in June (19.3–21.7°C) and October (17.3–18.3°C) and at Peter’s Neck in late May—early June (15.8–20.6°C). At the East Marion ‘walking’ site, only limited walking behavior was observed in 2013 and 2015 and was not seen at all in 2014 and 2016 (Table 1).

### Characterization of hard clam walking behavior

**Fig 1:**
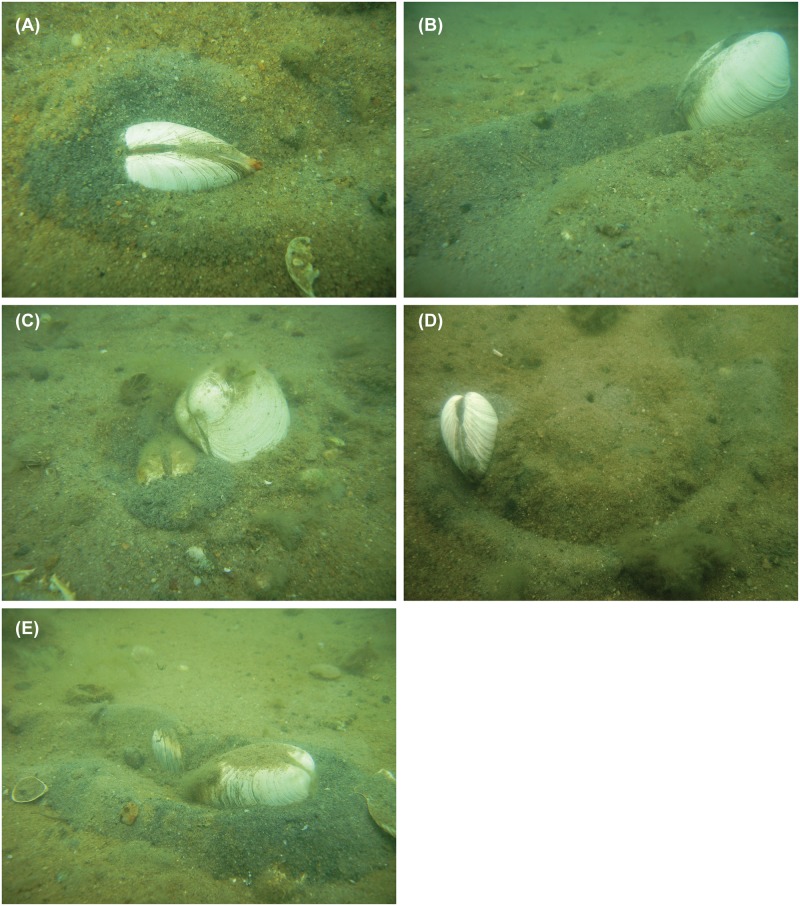
Hard clam, *Mercenaria mercenaria*, walking at East Marion, New York, USA. (A) Active walker illustrating typical shell orientation: ligament parallel to sediment surface, siphon above sediment in furrow. (B) Active walker with freshly excavated trail (furrow). (C) Non-walking clam lying on sediment surface, next to active walker. (D) Active walker at end of semi-circular trail. (E) Epibenthic clams, with posterior ends (where siphons are located) adjacent to one another.

Actively walking clams always moved in the anterior direction; most of those for which trails and shell height above the sediment were measured were epibenthic (n = 50/59), with the external, posterior ligament approximately parallel to the sediment surface and the siphon positioned above the sediment in the furrow behind the clam (Fig 1A and 1B). Occasionally, the foot was also visible. Often, recently excavated, black (anaerobic) sediments were seen at the leading (anterior) end of the clam. Non-walking clams were sometimes seen in the epibenthic walking position, but were almost always endobenthic, with the ligament perpendicular to the sediment surface and siphons pointed upward (i.e. the typical position for buried clams).

The mechanics of walking were very consistent: at the initiation of a walk, the clam tilted forward so the umbo moved closer to the sediment while the siphons were raised ~1 cm higher; this was accompanied by 2 consecutive releases of water from beneath the sediment, at the location of the lunule and likely from the mantle cavity, which caused sediment plumes to appear for < 1 s (S1 Video). Next, the clam moved forward, very likely pulled by the contraction of the extended foot below the sediment surface, as the shell rotated back to its original horizontal position; this was also accompanied by another release of water (S1 Video). Both siphons appeared to remain open during the walking process, perhaps even during shell rotation/water release. The mean duration of 32 recorded walks was 2.4 sec (standard error of the mean, SE = 0.05); the time interval between walks ranged from 68–184 s (mean = 85.7 s; SE = 5.6) (S1 Table). The distance traveled in a given walk was estimated to be 2–4 mm.

Shell height above the sediment surface in actively walking clams ranged from 0–60 mm (mean = 25.1 mm; SE = 0.21) while furrow depth behind recently or actively walking was very similar (mean = 24.5 mm; SE = 0.12): S2 Table. Shell length had no effect on either of these respective metrics in Ancova’s (F = 0.342, p = 0.561 and F = 0.914, p = 0.344); subsequent 1-way Anova’s showed that clam gender had no effect on shell height above the sediment surface or furrow depth (F = 1.368, p = 0.247 and F = 1.058, p = 0.309).

Trails made by walking clams were 5–50 cm in length; trajectory of walking clam trails (n = 82) ranged from linear (59.8%) to curved (13.4% to the left, 15.8% to the right) to S or Z-shaped (6.1%) to semi-circular (4.9%): Fig 1D, S2 Table. Trail length did not vary (t = 0.07; p = 0.947) between seasons (June/July: mean = 19.2 mm, n = 98; October: mean = 19.5 mm, n = 4). Shell length had no effect on trail length in an Ancova (F = 0.263, p = 0.610; a subsequent 1-way Anova showed that clam gender had no effect on trail length (F = 1.339, p = 0.251. There was no statistical evidence for directionality of clam heading (χ^2^ = 11.23, p > 0.10, n = 79).

### Maximum duration of epibenthic habitation

Several clams were also observed lying on the sediment surface, sometimes touching (Fig 1C); these showed no evidence of trails. Some of these clams had epifaunal *Crepidula fornicata*, up to 46 mm SL. Using the growth formula of [36], age of the largest *C*. *fornicata* was calculated to be 7.52 yr; thus, clams may have lain on the sediment surface for this long. However, our observations (S. Tettelbach et al., unpub data) of comparable sizes of *C*. *fornicata* epibionts on adult (~16–17 mo old) bay scallops, *Argopecten irradians irradians*, suggest much faster growth rates of this species in the Peconic Bays, New York and, hence, shorter duration of *Mercenaria* lying on the sediment surface.

### Comparison of ‘walking’ and ‘non-walking’ areas

#### Environmental factors

There were no differences in total chlorophyll a or the ≥5 μm chl a size fraction in phytoplankton samples from the ‘walking’ and ‘non-walking’ areas at East Marion: Mann-Whitney T = 42.00, p = 0.699; t = 0.15, p = 0.882, respectively (S3 Table). There was no difference in the proportion of sand in surficial sediment samples from the two areas (t = 0.80, p = 0.435: S4 Table). Temperatures measured at the ‘walking’ and ‘non-walking’ areas on the same days were identical (Table 1).

#### Clam gender and condition

Gender frequency did not differ amongst actively walking (67 males, 50 females: χ^2^ for equal gender frequency = 2.48; p > 0.05: S5 Table) or non-walking clams (46 males, 53 females: χ^2^ for equal gender frequency = 0.36; p > 0.50: S6 Table). Shell length of these 216 clams did not differ with respect to gender (F = 1.87, p = 0.173) or area (‘walking’ vs. ‘non-walking’: F = 0.41, p = 0.522), but there was a slight gender x area interaction effect (F = 4.66, p = .032).

Histological analyses revealed that 20 (13 males, 7 females) of 23 walking clams sampled on 19 July 2010 were in spawning condition; all but one were in early-mid spawning state (S4-S2: S5 Table). Of 28 clams sampled on 12 June 2012, 10 of 16 in the ‘walking’ area were in stages S4-S2 while 8 of 12 clams in the ‘non-walking’ area were in spawning condition (5 in stages S4-S2). There was no difference in the frequencies of clams in stages S4-S1 in the ‘walking’ and ‘non-walking’ areas: Fisher Exact Test: p > 0.50.

Only two of 51 clams (both from the ‘walking’ area) sampled on the 2 dates had any parasitic infection and these were very light: only 1 trematode in visceral mass and 1 in gill tissue of different animals (S5 Table). There was no difference in parasite frequency in clams from the ‘walking’ and ‘non-walking’ areas: Fisher Exact Test: p = 1.0. Gonadal smears of 3 female clams on 12 October 2011 revealed that all were reproductively active.

#### Population parameters

**Fig 2:**
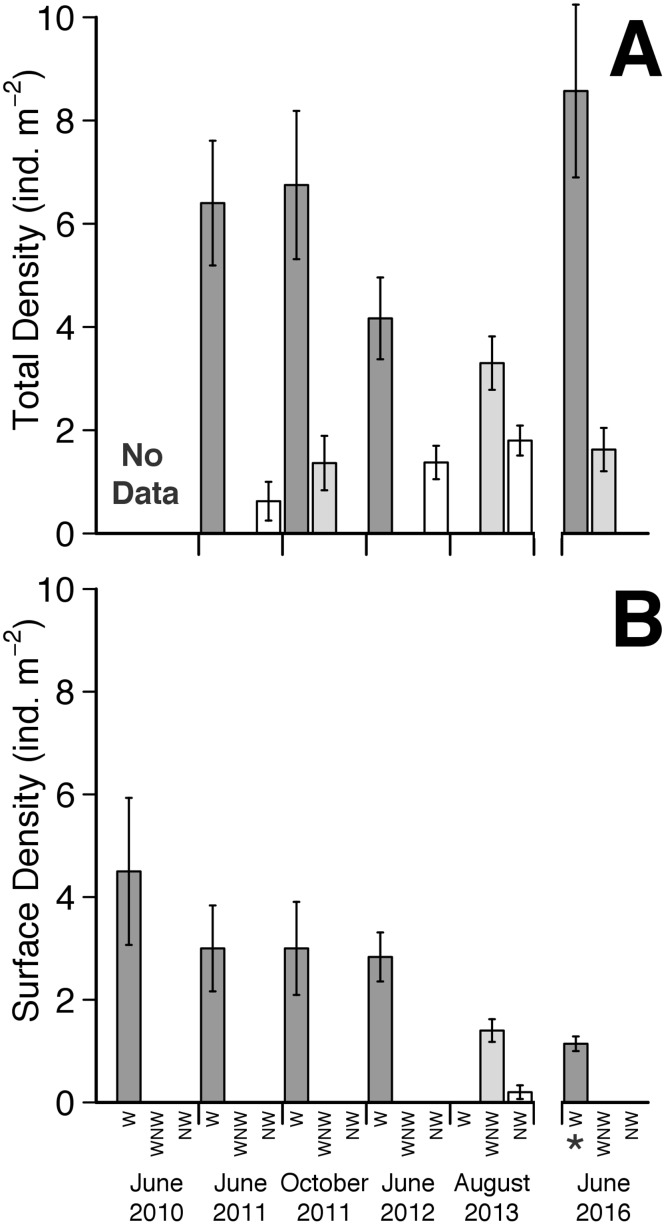
Bar plots of mean (±1 standard error of the mean, SE) *Mercenaria mercenaria* density versus site/walking designation in Orient Harbor, New York, USA. (A) Total (epibenthic + endobenthic) clam density. (B) Surface (epibenthic) clam density. All densities quantified at the East Marion site, except where noted as * (= Peter’s Neck site). W = walking clams, in ‘walking’ areas; NW = non-walking clams, in ‘non-walking’ area; WNW = non-walking clams in ‘walking’ area.

Total (epibenthic + endobenthic) clam densities differed dramatically (F = 11.88, p < 0.001) between areas, with densities significantly (t > 3.36, p < 0.05) higher among walking clams (in the ‘walking’ area) at East Marion in 2011–2012, at Peter’s Neck in 2016, and among non-walking clams at the East Marion ‘walking’ area in 2013 versus: non-walking clams in (1) the ‘non-walking’ area at East Marion (2011–2013) and (2) the ‘walking’ area at East Marion (2011, 2016) (Fig 2A, S7 Table). Similarly, epibenthic clam densities were significantly higher (Kruskal-Wallis H = 76.02, p < 0.001; Dunn’s Q > 3.36, p < 0.05) in the ‘walking’ area at East Marion in 2010–2013 compared to areas where no clam walking was observed in 2011–2013 and 2016 (Fig 2B, S7 Table). The highest numbers of clams observed to be actively walking (Table 1) always corresponded to the highest overall mean clam densities (4.17–6.75 m^-2^ at East Marion, 8.57 m^-2^ at Peter’s Neck) (Fig 2A). No active walking at East Marion was observed at lower overall mean clam densities (0.62–3.3 m^-2^).

As determined in quadrats centered on actively walking individuals at the East Marion ‘walking’ site, in June, epibenthic and walking *Mercenaria*, respectively, comprised 47% and 16% (2011) and 68% and 24% (2012) of respective populations (S7 Table). In 2016, respective numbers were both 0% in June at the East Marion ‘walking’ site. At Peter’s Neck, in late May—mid-June 2016, they were both 13%.

**Fig 3:**
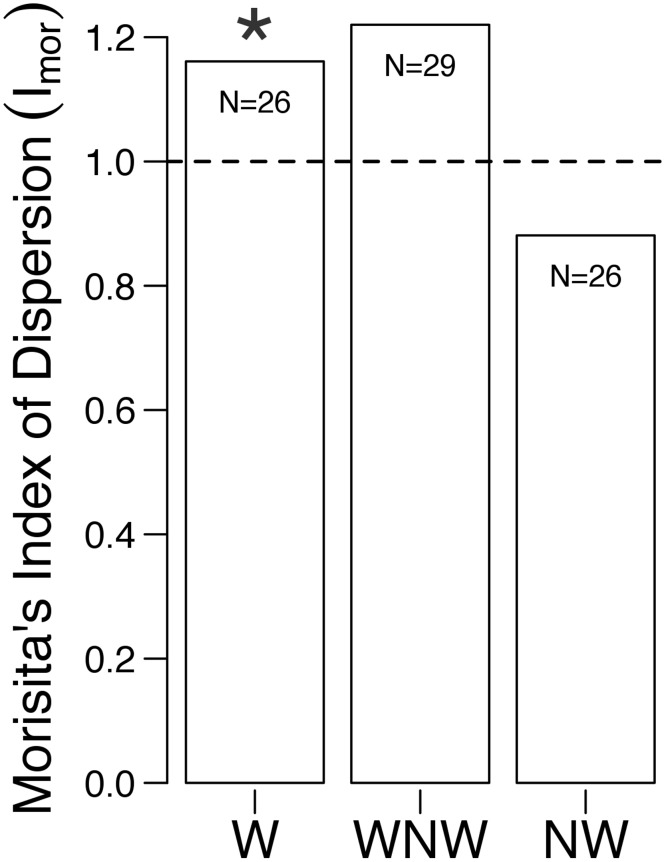
Morisita’s Index for *Mercenaria mercenaria* versus site/walking designation in Orient Harbor, New York, USA. W = walking clams, in ‘walking’ areas; NW = non-walking clams, in ‘non-walking’ area; WNW = non-walking clams in ‘walking’ area. Dashed line demarcates transition from a random (I_M_ = 1) to an aggregated distribution (I_M_ > 1); asterisk denotes a significant χ^2^ test: p = 0.001.

Clams in the ‘walking’ area at East Marion were significantly aggregated (composite I_M_ = 1.161, p < 0.001, n = 26) (Fig 1E) while those in the ‘non-walking’ area (n = 26, composite I_M_ = 0.881, p = 0.689), as well as the non-walkers in the ‘walking’ area (n = 29, composite I_M_ = 1.220, p = 0.051), were more dispersed, although not statistically different from random (Fig 3).

**Fig 4:**
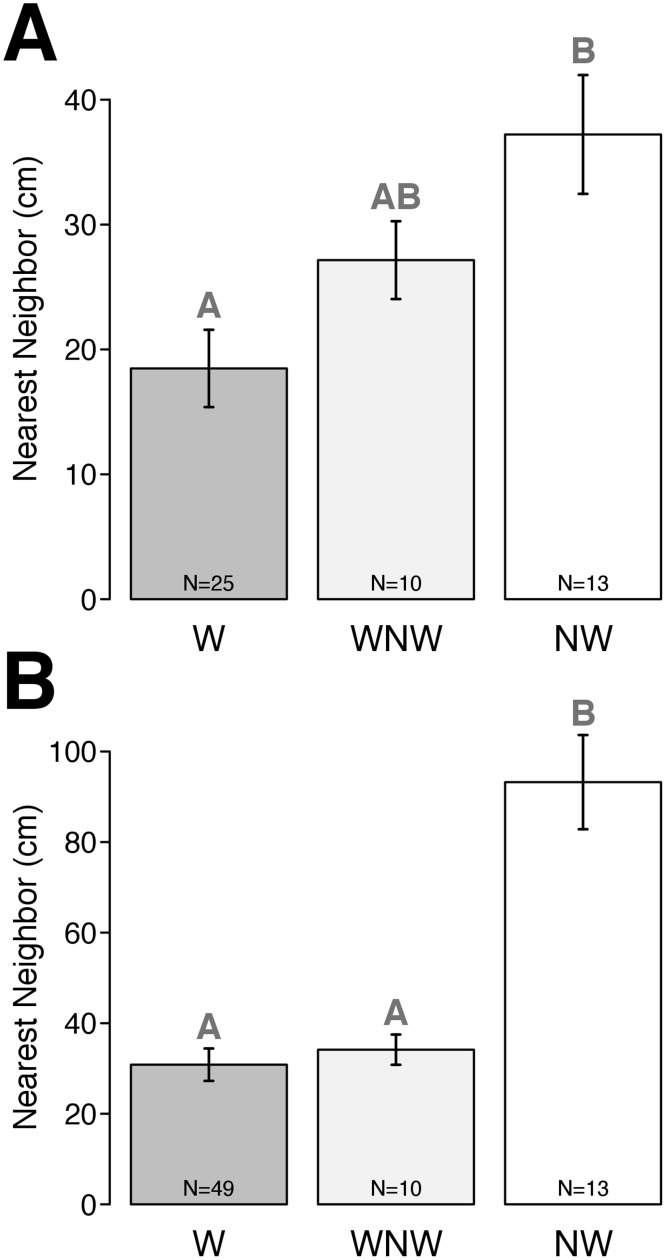
Bar plots of mean (±1 standard error of the mean, SE) nearest neighbor distances of *Mercenaria mercenaria* at East Marion, New York, USA. (A) endo- + epibenthic clams and (B) epibenthic clams only. W = walking clams, in ‘walking’ area; NW = non-walking clams, in ‘non-walking’ area; WNW = non-walking clams in ‘walking’ area. Different letters above bars represent significantly different densities at p < 0.05.

When epi- and endobenthic clams were considered together, mean distance of actively walking clams to their nearest neighbor (18.5 cm) was significantly less (F = 7.64, p = 0.001) than that for clams in the ‘non-walking’ area at East Marion (37.2 cm); mean nearest neighbor distance for non-walking clams in the ‘walking’ area was intermediate (27.2 cm) (Fig 4A, S8 Table). For epibenthic clams, mean NN distances for walking (30.7 cm) and non-walking clams in the ‘walking’ area (36.3 cm) were significantly less (F = 12.07, p < 0.0001) than for clams in the ‘non-walking’ area (58.6 cm) (Fig 4B, S9 Table).

Analysis of the genders of actively walking hard clams and their respective single nearest epibenthic neighbor, in any direction from the active walker were not different from a 1:1 sex ratio (χ^2^ = 1.52, p > 0.20, n = 33: S10 Table). However, a similar analysis for epibenthic gender pairs in the forward 120° direction of actively walking individuals revealed that clams were significantly more likely to move toward a member of the opposite sex (χ^2^ = 4.08, p < 0.05, n = 12: S11 Table).

## Discussion

### Walking behavior of hard clams and other bivalve mollusks

Hard clam walking is not a rare phenomenon in the Peconic Bays, New York, USA as it was observed at 9 of 15 different sites where we noted epibenthic hard clams. Furthermore, this behavior was exhibited by sizes of *Mercenaria* much larger than previously reported. So, why has this phenomenon not been previously described? Perhaps this is a case of being in the right place at the right time to observe active clam walking and/or requires a specific combination of sediment composition, depth, current regime, water temperature and clam density. Nevertheless, hard clam densities above those at which we observed extensive walking have been commonly cited in the literature [34]. Spatial and temporal patterns of *Elliptio complanata* emergence in lakes were found to vary markedly with substrate and levels of physical disturbance; i.e. wind fetch [51]. A significant positive correlation was also noted between the numbers of epibenthic *E*. *complanata* exhibiting horizontal movement and day length; clams began moving in mid-May, with the proportion of walking individuals increasing rapidly until July, but declining quickly afterward [14]. The mean daily distance traveled by walking *E*. *complanata* was greatest during the spawning season, with a peak in June/July [14]. Our finding of the highest levels of walking activity in June/July and October (with less during the months between), also suggests that hard clam walking is less correlated to absolute water temperature than seasonal spawning activity—which was prominent in June/July and likely in progress during October [44]. However, we saw equal *Mercenaria* trail lengths during the two periods, albeit with a small sample size in the latter period. The apparent, but unexplained reduction in numbers of epibenthic hard clams in mid-summer was also noted for *E*. *complanata* [15].

Despite the dramatic and readily recognizable walking behavior, it was not clear why more hard clams did not exhibit this behavior. Lotic *E*. *complanata* less than 3 yr were always endobenthic, but up to 80% of older mussels were epibenthic [52]. [14] found that an average of only 2–8% of lentic *E*. *complanata* moved in ice-free seasons. Given the high numbers of epibenthic *Mercenaria* that we observed without trails, we conclude that the adoption of the walking position does not necessarily progress to active walking; clam density may be key in this regard (see below).

Besides *Mercenaria* and *E*. *complanata*, walking has been described for numerous other species of Unionids, e.g. [16,17,53] and *Tridacna squamosa* [54]. Active walking by another venerid clam, probably *Lioconcha castrensis* (at Dravuni, Fiji in 1999), and by *Spisula solidissima* (at the Gardiners Bay site on 1 June 2007) have also been directly observed by STT. Thus, walking behavior may be more common amongst bivalves than is generally realized.

### Characterization of walking behavior

As determined via video analysis, the mechanics of *Mercenaria* walking at the sediment surface seem to be very similar to those described for vertical digging by this species [55] and several other bivalves, including *Macomangulus* (*Tellina*) *tenuis*, *Donax vittatus*, *Cerastoderma* (*Cardium*) *edule* and *Limecola* (*Macoma*) *balthica* [56], *Venerupis corrugata* (formerly *pullastra*) [57] and *Anodontites trapesialis* [53]. The siphons of the 4 species examined by [56] remained open during the digging cycle until just before water was ejected from the mantle cavity, at which time they closed for a brief period. Our observations suggest that hard clam siphons remain open during walking, perhaps even at the time of water release (from the mantle cavity).

### Underlying causes and potential costs/benefits of hard clam walking

Hard clam walking may represent either a stress response or an adaptive behavior to increase the probability of survival, growth or reproduction. We saw no evidence this behavior was induced by parasites, as in *Limecola* (*Macoma*) *balthica* [27]. Juvenile *Mya arenaria* were observed to exhibit less burrowing and higher dispersal in sediments that were experimentally acidified [58]. However, if hard clam walking was a response to low pH sediments or some other type of stress (e.g. crowding, food limitation, discharge of toxins from groundwater) then this behavior might be expected to lead to decreased, rather than increased aggregation. In comparing ‘walking’ and ‘non-walking’ areas at East Marion, we saw no differences in clam size or gender composition, frequency of individuals in spawning condition, parasite load, temperature, chl a levels in the water column (although these would not be expected to vary much given the proximity of the two areas) or sediment grain size. Similarly, no correlation was found between distribution of lotic *E*. *complanata* and sediment type [52]. The only significant differences between the two East Marion sites were the shorter nearest neighbor distances, higher density and higher degree of aggregation of hard clams in the ‘walking’ versus ‘non-walking’ area.

Aggregation represents a trade-off between the costs and benefits of close proximity [54]. The process of walking at the sediment surface potentially exposes bivalves to higher predation [15,17]. However, *Mercenaria* of the sizes we observed are greater than those which might be successfully attacked by the majority of non-human predators in the Peconic Bays [36,59]. While channeled (*Busycotypus canaliculatus*) and knobbed whelks (*Busycon carica*) are both common at the sites where walking was observed [36], feeding rates of these species on *Mercenaria* are low [60]. Aggregation may increase the probability of parasite transmission or intraspecific competition [27,61], but observed parasite loads in sampled clams were very low. Potential benefits of bivalve aggregation may include increased survival due to reduced rates of predation, increased stabilization of the physical environment [62] or increased reproductive success [21–24]. Walking at the sediment surface by Unionids [15,23] and by *Tridacna squamosa* [54] is believed to serve the purpose of aggregating reproductively mature individuals in preparation for spawning. Furthermore, the spatial distribution of *E*. *complanata* has been found to change from highly aggregated just prior to spawning to near random following spawning [15]. The probability of successful fertilization of eggs increases when spawning individuals are closer together [21–24]; population modeling conducted for *Mercenaria mercenaria* in Great South Bay, New York suggests that the maximum distance between spawning individuals needs to be ≤ 0.75 m to ensure fertilization [25].

Our observations suggest that the greatest levels of hard clam walking activity occurred just before/while clams were in peak spawning condition. Given that actively walking clams also were shown to be significantly more likely to move toward a member of the opposite sex, we conclude that *Mercenaria* are walking to get closer to one another prior to spawning.

### Proximate cues for induction of hard clam walking

Given that the highest numbers of walking *Mercenaria* were always observed in areas with the highest densities, it is apparent that some threshold density is necessary to cue extensive walking behavior. As compared to the ‘walking’ area at East Marion, where extensive hard clam walking was observed from 2010–2012, walking at the ‘non-walking’ area was observed just twice (and only by ~5 clams); as discussed above, the only noted differences between the two areas were the greater nearest neighbor distances, lower density and lower degree of aggregation in the ‘non-walking’ area. Furthermore, the lack of extensive walking at the East Marion ‘walking’ site in later years of the study appears to reflect decreased clam densities: 3.3 m^-2^ in 2013 and 1.63 m^-2^ in 2016 vs 4.17–6.75 m^-2^ in 2011–2012. This again suggests that extensive walking only occurs above some threshold density, perhaps >3.5–4 clams m^-2^.

The specific stimuli that induce hard clam walking may be chemical, as confirmed for *Tridacna squamosal* [54]. If the cue for walking is some type of pheromone, e.g. as found in sperm of the oyster, *Crassostrea virginica* [63], then increased walking activity should be expected at higher pheromone concentrations and/or lower dilution. Hard clam walking was most commonly seen at sites (East Marion, Peter’s Neck) with high clam densities and low current flow—both of which should increase the probability of chemical detection. The above conclusion is also supported by the lack of directionality of clam walking along the primary axes of tidal flow, also as noted by [28]. [52] observed erratic horizontal movements in *Elliptio complanata*; tagged individuals in a lotic population moved an average distance of 2.9 m in 1 yr, but only showed a net downstream movement of 27 cm. Males and females of two marine copepod species were shown to react to pheromones, where swimming behaviors had stochastic properties that were dependent on pheromone concentration, sex and species, as well as reproductive experience at the level of the individual [64]. These authors [62] concluded that copepod swimming behaviors were consistent with an adaptation to increase the rate of encounter with prospective mates and thus for optimizing reproductive success and fitness. Our investigations of hard clam walking, including our finding that walking clams were significantly more likely to move toward a member of the opposite sex, suggest interesting parallels to the work of [64], but further experimental study is needed to investigate the importance of pheromones and other environmental factors as drivers of this behavior in *Mercenaria*. Another potential factor which would be interesting to investigate in the context of mate detection, and thus in cuing walking behavior, is whether hard clams can detect pressure waves generated by infaunal movements—which have been shown to be both species- and activity-specific and were detectable at distances of ≥20 cm [65]. Interestingly, mean trail length of active walkers in the ‘walking’ area, from 2010–2012, was 19 cm, while mean distance of actively walking clams to their nearest neighbor (endo- or epibenthic) was 18.5 cm.

If extensive walking does not occur below a threshold density, it is logical to conclude that cue(s) are either absent or not strong enough to elicit this behavior at low densities. If the energetic cost of walking toward another individual is high, clams may not progress from the epibenthic walking position (which was seen frequently) to actual walking (which was seen much less frequently), and continue this behavior for any length of time—unless there is some reinforcement, perhaps through sensing an increase in cue factor levels produced by conspecifics.

These are interesting topics, in their own right, for further investigation, but they also allude to broader questions about the comparative benefits and mechanisms of aggregation in sedentary species: in low versus high density populations of one species or in rare versus common species. Fertilization success in many species is dependent on both population density and population size [21,22,24,66] and thus the largest proportional benefit of aggregating to increase fertilization success should theoretically occur at very low population densities. However, Allee effects (see below) may negate such potential benefits. Strong self-limitation has been demonstrated to promote the persistence of rare species [67]; further investigation is needed to explore the specific mechanisms, including behavioral and physiological ones [68], by which fertilization may be facilitated in species with low density populations [69].

### Apparent suppression of walking behavior by oversampling

The year to year reduction in hard clam density and walking behavior in our ‘walking’ area seems to have resulted, at least in part, from the permanent removal of >200 adult clams for scientific analyses. The legacy of our presumed oversampling (overharvesting) seems to be a lack of extensive hard clam walking at the East Marion ‘walking’ site for >3 years; very low numbers of juvenile *Mercenaria* observed here may mean the lack of walking persists for a much longer period.

There are many examples whereby intensive fishing activities have strongly impacted the biology and ecology of aquatic species, such as altered life history characteristics (e.g. earlier age at first reproduction in several North Atlantic and Great Lakes finfish species) [70] or Allee effects at lower population densities, resulting in reduced mating frequency in queen conch, *Lobatus gigas* [71] and likely reduction of fertilization success in the unionid *Elliptio complanata* [23] and sea scallop, *Placopecten magellanicus* [72]). Overharvesting has also driven species extinctions (e.g. Steller’s sea cow, *Hydrodamalis gigas*: [2]) and, rarely, scientific collecting (e.g. for voucher specimens) has been implicated in contributing to extinctions of endangered species [73].

In our investigation of a previously unknown behavior, exhibited by an important commercial species, we unknowingly appear to have suppressed a behavior whose likely purpose is to increase the probability of fertilization success. This inadvertent impact to a population of *Mercenaria mercenaria* illustrates that heightened levels of scientific awareness and caution are warranted during research activities, even while conducting novel studies on the biology and ecology of commercially exploited species.

## Conclusions

We have described hard clam walking, a locomotory behavior of a very well-studied species that does not appear to be rare, yet whose mention had been almost non-existent in the literature. This behavior, which appears to be induced above some threshold density (perhaps >3.5–4 clams m^-2^) and may be mediated by unidentified pheromone(s), pressure waves and/or other unidentified factors, serves to aggregate mature individuals prior to spawning and, thereby, presumably facilitates greater fertilization success. Further study is needed to confirm the proximate causes and full significance of hard clam walking. In the process of investigating this behavior, we apparently reduced clam densities below the minimum density threshold and, in so doing, suppressed the behavior for a period of >3 years running. This reinforces the importance of detailed field investigations of species biology and ecology, even for those that are considered to be well known, but also highlights the need for greater awareness of the potential impacts of scientific research activities.

## Supporting information

S1 Video Hard clam walking. June 2010.
(MP4)

S1 Table Hard clam walking: Duration, interval between walks. Video data.
(XLS)

S2 Table Characterization of hard clam walking behavior. Active walkers.
(XLSX)

S3 Table Chlorophyll *a* data. (XLSX)

S4 Table Surficial sediment data. (XLSX)

S5 Table Reproductive condition, parasite load of walking, non-walking hard clams. Histological data.
(XLSX)

S6 Table Sizes, genders of non-walking hard clams. (XLSX)

S7 Table Hard clam density data. (XLSX)

S8 Table Distance to nearest neighbor: Epi- or endobenthic hard clams. (XLSX)

S9 Table Distance to nearest neighbor: Epibenthic hard clams. (XLSX)

S10 Table Gender pairs of actively walking hard clams and their single nearest epibenthic neighbor, in any direction. (XLSX)

S11 Table Gender pairs of actively walking hard clams and their single nearest epibenthic neighbor, in 120° forward direction. (XLSX)
